# Distinct neuronal mechanisms for motor impairment and seizures in a novel mouse model of *SCN8A* epileptic encephalopathy

**DOI:** 10.1016/j.nbd.2026.107317

**Published:** 2026-02-10

**Authors:** Midhun N.K. Anne, Laura Kakuk-Atkins, Jason Kaplan, Adam S. Deardorff, Meretta A. Hanson, Aidan C. Johantges, Alec H. Marshall, Stephen J. Kolb, Jason C. Wester, Jacy L. Wagnon

**Affiliations:** a Department of Neuroscience, College of Medicine, The Ohio State University, Columbus, OH 43210, USA; b Molecular, Cellular, and Developmental Biology (MCDB) graduate program, The Ohio State University, Columbus, OH 43210, USA; c Department of Clinical Neurosciences; Wright State University Boonshoft School of Medicine; Dayton, OH 45435, USA; d Department of Neuroscience, Cell Biology, and Physiology; Wright State University Boonshoft School of Medicine; Dayton, OH 45435, USA; e Department of Neurology, The Ohio State University, Columbus, OH 43210, USA; f Neuroscience graduate program, The Ohio State University, Columbus, OH 43210, USA; g Department of Biological Chemistry & Pharmacology, The Ohio State University, Columbus, OH 43210, USA.

**Keywords:** Epilepsy, Developmental and epileptic encephalopathy, Movement disorder, Nav1.6, Sodium channel, Ion channel

## Abstract

Variants in the voltage-gated sodium channel gene *SCN8A* cause a severe developmental and epileptic encephalopathy (DEE) characterized by treatment-resistant seizures, developmental delay, long-term cognitive and motor impairment, and elevated risk of premature death. The most common comorbidity is motor impairment, including hypotonia, movement disorders like ataxia, and weakness. To date, mouse models of *SCN8A* DEE have recapitulated seizures and early death, but have not exhibited motor impairment. We developed a novel conditional mouse model of *SCN8A* DEE with the patient mutation p.Thr767Ile (T767I). Ubiquitous expression of the T767I allele with Sox2-Cre (*Scn8a*^T767I/+^) results in neuronal hyperexcitability, spontaneous convulsive seizures, and premature death in heterozygotes. *Scn8a*^T767I/+^ mice also exhibit significant early-onset motor impairment and muscle weakness. Mice with expression of the T767I allele in excitatory neurons driven by Emx1-Cre experience seizures and early death but do not exhibit motor impairment, indicating that the neuronal mechanisms underlying seizures are distinct from the mechanisms underlying motor impairment. Compound muscle action potentials are smaller, and the number of functional motor units is reduced in Sox2-Cre, *Scn8a*^T767I/+^ mice, suggesting that motor neuron function is affected by the T767I mutation. Neuromuscular junctions exhibit morphological abnormalities and appear to have delayed maturation in *Scn8a*^T767I/+^ mice compared to *Scn8a*^+/+^ mice. The *Scn8a*^T767I/+^ mouse is the first model of *SCN8A* DEE to recapitulate motor impairment. This novel mouse model will permit elucidation of the pathogenic mechanisms underlying motor impairment in *SCN8A* DEE.

## 1. Introduction

Developmental and epileptic encephalopathies (DEEs) are severe neurological disorders characterized by seizures along with significant developmental delay and/or regression. Variants in the voltage-gated sodium channel gene *SCN8A* cause DEE with treatment-resistant seizures and comorbidities including motor impairment, cognitive impairment, and elevated risk of early mortality (Scheffer et al., 2017). Low muscle tone, physical weakness, and movement disorders (e.g., ataxia, myoclonus, dystonia, dyskinesia, and choreoathetosis) are the most prevalent comorbidities in *SCN8A* DEE, affecting more than 75% of patients and greatly impacting quality of life (Encinas et al., 2019; Talwar and Hammer, 2021). Approximately 50% of children with *SCN8A* DEE do not develop the ability to walk or sit properly. Identifying the pathophysiological mechanisms underlying motor impairment in *SCN8A* DEE is important for understanding the role of *SCN8A* in normal and abnormal motor function, as well as for developing therapeutic strategies that address comorbidities like motor impairment.

More than 400 variants have been identified in *SCN8A* DEE and other *SCN8A*-related neurological disorders (Johannesen et al., 2021; Talwar and Hammer, 2021). Functional analyses carried out on approximately 15% of these variants demonstrate diverse biophysical effects on Na_v_1.6 function. *SCN8A* DEE is caused by missense variants that result in primarily gain-of-function (GOF) effects, including altered channel activation, impaired channel inactivation, and abnormal persistent, ramp and resurgent currents (Estacion et al., 2014; Johannesen et al., 2021; Y. Liu et al., 2019; Pan and Cummins, 2020; Solé et al., 2020; Tidball et al., 2020; Veeramah et al., 2012; Wagnon et al., 2016; Zaman et al., 2019).

Given the large number of missense variants identified in *SCN8A* DEE, and the heterogeneous GOF effects in these variants, multiple models are needed to define pathogenic mechanisms. To date, two mouse models of *SCN8A* DEE based on patient variants knocked-in to the endogenous locus have been characterized (Bunton-Stasyshyn et al., 2019; Wagnon et al., 2015). The *Scn8a*^N1768D/+^ mouse exhibited spontaneous seizures beginning as early as 2 months of age. *Scn8a*^N1768D/+^ mice progress to seizure-related sudden death within 30 days of seizure onset (Wagnon et al., 2015). The second model, a conditional allele of *Scn8a*^R1872W^, resulted in a severe phenotype with one terminal tonic seizure at P15 when expressed ubiquitously using E2a-Cre (Bunton-Stasyshyn et al., 2019). Although both models recapitulate seizures and premature lethality, the heterozygotes conspicuously lack motor dysfunction and do not differ from wildtype mice in righting reflex, gait, or muscle strength (Bunton-Stasyshyn et al., 2019; Wagnon et al., 2015). *Scn8a*^N1768D/N1768D^ homozygotes and *Scn8a*^N1768D/−^ mice exhibit tremor and ataxia. While not reflective of the heterozygous patient genotype, motor abnormalities in these mice that carry only the pathogenic N1768D allele provide evidence for the important role of Na_v_1.6 in motor function.

In contrast to mouse models of *SCN8A* DEE with GOF variants in *SCN8A*, several mouse lines with loss-of-function (LOF) variants of *SCN8A* have well-described motor phenotypes. Hypomorphic alleles of *Scn8a* with more than 50% reduction of Na_v_1.6 cause movement disorders in mice, including dystonia and ataxia (Jones et al., 2015; Meisler et al., 2001). Mice with complete loss of Na_v_1.6 exhibit failure and degeneration of the neuromuscular junction (NMJ) that precedes muscle paralysis (Kearney, 2002; Meisler et al., 2001). From these data, it is clear that Na_v_1.6 plays an important role in motor physiology. However, the effects of GOF mutations on motor physiology have not been examined, largely due to the lack of motor phenotypes in the two existing mouse models with GOF *Scn8a* mutations (N1768D and R1872W).

Na_v_1.6 is the major neuronal ion channel at axon initial segments and mature nodes of Ranvier in mammals. In addition to the CNS, *SCN8A* is expressed in motor neurons and in parvalbumin-positive proprioceptive sensory neurons (Duflocq et al., 2008, 2011; Espino et al., 2022). To interrogate the neuronal circuits underlying motor comorbidities in *SCN8A* DEE, we developed a new conditional mouse model with the GOF patient variant p.Thr767Ile (T767I). *Scn8a*^T767I/+^ mice recapitulate the seizures and premature lethality characteristic of *SCN8A* DEE. Hippocampal CA1 neurons are hyperexcitable in *Scn8a*^T767I/+^ mice, consistent with phenotypes observed in the previous mouse models. Importantly, *Scn8a*^T767I/+^ mice also demonstrate significant motor impairment, permitting investigation of the pathophysiology of motor impairment in *SCN8A* DEE for the first time. Restriction of expression of *Scn8a*^T767I^ to forebrain excitatory neurons using Emx1-Cre is sufficient to generate seizures without motor impairment, indicating that the full clinical *SCN8A* DEE phenotype with seizures and motor impairment involves GOF Na_v_1.6 activity in other types of neurons. Here, we demonstrate that compound muscle action potentials are smaller in *Scn8a*^T767I/+^ mice. The number of motor units, consisting of motor neurons and innervated skeletal muscle, are reduced in *Scn8a*^T767I/+^ mice, indicating that motor neuron activity is impaired by the T767I variant. Neuromuscular junctions (NMJs) are smaller with reduced branching complexity and decreased synaptic contact, suggesting that NMJs are dysfunctional in *Scn8a*^T767I/+^ mice. Taken together, our data demonstrate that seizure activity and motor impairment are caused by alterations in *Scn8a* activity in distinct neuronal subtypes. In addition, our results suggest that motor unit dysfunction contributes to motor impairment in SCN8A DEE.

## 2. Materials and methods

### 2.1. CRISPR/Cas9 mediated genome targeting

Genome targeting was carried out by the custom model generation service at The Jackson Laboratory. CRISPR/Cas9 was used to knock-in a lox-stop-lox cassette upstream of the p.Thr767Ile mutation in exon 14. Residue Thr767 in human is equivalent to residue Thr765 in mouse, and we have elected to keep the Thr767 for clarity and consistency with the two previous mouse models of *SCN8A* encephalopathy. The lox-stop-lox inhibits constitutive expression of the *Scn8a*-T767I allele. Exposure to CRE recombinase restores expression of the T767I allele, generating *Scn8a*^T767I/+^ heterozygosity in any *Cre*-expressing cells. Targeted mice were generated on the C57BL/6J genetic background, thus no backcrossing was needed to establish a congenic line. Correct targeting was verified by long-range PCR and sequencing. Two founder mice were obtained and were bred with C57BL/6J mice to generate N1 progeny. We maintain the targeted allele, *Scn8a*^cond^, by breeding *Scn8a*^cond/+^ mice to C57BL/6J mice in the absence of CRE, thus protecting against an early lethal phenotype and preserving a congenic strain background. This cross also generates *Scn8a*^+/+^ wildtype littermates used for experiments.

### 2.2. Animals

All mice were housed and cared for in accordance with NIH guidelines in a 12/12-h light/dark cycle with standard moue chow and water available ad libitum. Experiments were approved by the Institutional Animal Care and Use Committee at The Ohio State University. *Scn8a*^cond/+^ mice heterozygous for the conditional allele were crossed with the Cre lines *Sox2-Cre* (JAX 008454) or *Emx1-Cre* (JAX 005628). The *Sox2-Cre* transgene is expressed in epiblast cells at embryonic day 6.5 and results in expression of the *Scn8a*^T767I/+^ genotype in all neurons(Hayashi et al., 2002). The *Emx1-Cre* transgene is expressed in precursors to glutamatergic forebrain neurons(Gorski et al., 2002) and was used to restrict expression of the *Scn8a*^T767I/+^ genotype to forebrain excitatory neurons. The total number of animals used in this study was 334.

### 2.3. Genotyping assays

Mice carrying the *Scn8a*^cond^ allele were routinely genotyped by PCR using a forward primer with sequence 5’-TGGTGGGGATCTTAACTCGG-3′ and a reverse primer with sequence 5’-GCGACTTCAGAGTTCTGCAG-3′ followed by digestion with BciIV to generate products of 713 bp and 298 bp for the wildtype allele, 1745 bp and 311 bp for the conditional allele, and 1003 bp for the activated T767I allele.

### 2.4. RNA extraction and Quantitative RT-PCR

Total RNA was isolated from whole brain by TRIzol extraction. cDNA was synthesized using LunaScript RT SuperMix Kit (NEB). TaqMan gene expression assays were performed using the TaqMan Gene Expression Master Mix. Reactions were run in quadruplicate. Total *Scn8a* transcript was assayed using FAM-labelled gene expression assay Mm00488119_m1 standardized to the VIC-labelled *Tbp* endogenous control Mm01277042_m1. Relative transcript quantity was calculated using the ddCT method (Livak and Schmittgen, 2001).

### 2.5. Whole-cell patch clamp electrophysiology

Whole-cell patch clamp physiology in brain slices was performed as previously published (Hanson et al., 2025). In brief, juvenile mice (PND14-PND17) were anesthetized with isoflurane and then decapitated. The brain was dissected in ice-cold artificial cerebrospinal fluid (ACSF) containing (in mM): 100 sucrose, 80 NaCl, 3.5 KCl, 24 NAHCO3, 1.25 NaH2PO4, 4.5 MgCl, 0.5 CaCl2, and 10 glucose, saturated with 95% O_2_ and 5% CO_2_. Coronal slices of dorsal CA1 hippocampus (300 μm) were cut using a Leica VT 1200S vibratome (Leica Microsystems) and incubated in the above solution at 35 °C for 30 min post-dissection. Slices were then maintained at room temperature until use in recording ACSF containing (in mM): 130 NaCl, 3.5 KCl, 24 NaHCO3, 1.25 NaH2PO4, 1.5 MgCl, 2.5 CaCl2, and 10 glucose, saturated with 95% O_2_ and 5% CO_2_. For recording, slices were constantly perfused with ACSF at 2 mL/min at a temperature of 31–33 °C. For recording, slices and cells were were transferred to an upright microscope (Scientifica SliceScope). Whole-cell patch clamp recordings were amplified using a Multiclamp 700B amplifier (Molecular Devices), filtered at 3 kHz (Bessel filter), and digitized at 20 kHz (Digidata 1550B and pClamp v11.1, Molecular Devices). Cells were recorded in current clamp and resting membrane potential (Vm) was biased to −70 mV. The internal solution contained (in mM): 130 K-gluconate, 5 KCl, 2 NaCl, 4 MgATP, 0.3 NaGTP, 10 phosphocreatine, 10 HEPES, 0.5 EGTA, and 0.2% biocytin. pH was adjusted to 7.4 with KOH. Recordings were not corrected for liquid junction potential. Series resistance (10–25 MΩ) was closely monitored throughout recordings. Recordings were discarded if series resistance passed 25 MΩ.

To measure active and passive membrane properties, constant amplitude current was injected in steps of 20 pA for 1 s duration. Voltage threshold for action potential initiation was calculated as the locus of the maximum of the second derivative of voltage as a function of time prior to a spike evoked at the rheobase current. Other measurements, including input resistance, voltage sag, and capacitance are as previously described (Hanson et al., 2025). All data were analyzed in Igor Pro (WaveMetrics) using custom routines.

### 2.6. Behavioral motor phenotyping

Body weight measurements were performed daily between PND 8 to PND 17. Motor phenotyping was performed as follows, modified from established protocols (Feather-Schussler and Ferguson, 2016). Gait was evaluated from PND 8 to PND 16. Walking behavior of each pup was observed in a clear cage without bedding for one minute. Gait score was given on a scale of 0–3, where score 0 indicates walking normally, score 1 indicates unevenness with mild limp/tremor, score 2 indicates severe limp/tremor without falling, score 3 indicates falling on a side completely, or falling on one quarter due to losing balance (ataxia) before being able to walk again. Righting time was measured from PND 8 to PND 10. Pups were placed on their backs in a supine position. The amount of time for a pup to return to its feet from the supine position was recorded, up to a maximum of 60 s. Forelimb suspension test was performed from PND 10 to PND 14. Pups were allowed to grab onto a metal wire strung across a cup with their forelimbs. Time spent hanging from the wire with only the forelimbs was recorded, up to a maximum of 60 s. Hindlimb suspension test was performed from PND 8 to PND 14. Pups were placed in a 50 milliliter tube and allowed to grasp the rim of the tube with their hindlimbs. The time taken for the pups to fall into the tube was recorded, up to a maximum of 60 s.

### 2.7. Muscle electrophysiology

Mice were anesthetized with isoflurane. Sciatic CMAP and MUNE responses were recorded from the right triceps surae while stimulating the sciatic nerve with single square-wave pulses of 0.1 msec duration as previously described (Arnold et al., 2015). Supramaximal CMAP responses were generated with stimulus currents <10 mA, and baseline-to-peak amplitude measurements were compared between mutant and control mice. Sciatic MUNE was performed with an incremental stimulus technique in which stimulus intensity was gradually increased to record a total of 10 incremental responses. The peak-to-peak amplitude of each individual motor unit response was calculated by subtracting the amplitude of the prior response. The 10 incremental values were averaged to estimate average single motor unit potential (SMUP) amplitude. The maximum CMAP amplitude (peak-to-peak) was divided by the average SMUP amplitude to yield the estimated number of motor units (MUNE).

### 2.8. Immunofluorescence of neuromuscular junctions

Mouse pups aged PND 15 were euthanized by CO_2_ followed by bilateral pneumothorax induction. Immediately after euthanasia, gastrocnemius muscle bundle from right hindlimb of each mouse was isolated and fixed in 10% formalin at room temperature for 48 h and stored in a solution of 30% sucrose and 0.1% sodium azide in phosphate buffered saline (PBS) at 4 °C. Muscle bundles were teased into 8–10 small bundles using fine tweezers under dissection microscope in PBS. Permeabilization of muscle bundles was performed by incubation in methanol at −20 °C for 10 mins. Then, muscles were blocked in freshly prepared blocking solution (1% bovine serum albumin and 0.5% Triton X-100 in PBS) for 1 h before incubation overnight at 4 °C on a shaker with 1:100 of each primary antibody (anti-2H3 and anti-SV2 antibodies; Developmental Studies Hybridoma Bank) in blocking solution. Muscles were washed three times (20 min each) with PBS followed by an overnight incubation at 4 °C on a shaker with 1:100 secondary antibody (Alexa Fluor^™^ 488 Goat anti-Mouse IgG; Invitrogen; cat# A11001) and 1:100 α-bungarotoxin, Alexa Fluor^™^ 647 (Invitrogen; cat# B35450). Muscles were washed three times (20 min each) with PBS. Muscle bundles were mounted with ProLong^™^ Gold antifade reagent (Invitrogen; cat# P36934).

### 2.9. Confocal Imaging and morphological analysis of neuromuscular junctions

Using a 63× oil objective lens, at least 15–25 confocal z-stack series of 1024 × 1024 frame size were taken, capturing one or multiple NMJs within each z-stack, on Leica SP8 confocal microscope. RGB images were captured with sequential image acquisition using two channels. Following image capturing, ImageJ was used for image processing. Initially, *en-face* NMJs were cropped with same length and breadth (square images, e.g. 192 × 192) from raw images and saved as maximum intensity projections (MIP). Then MIP image of each NMJ was converted into an 8-bit file after removing blue channel. The automated morphological analysis of each NMJ (square, 8-bit file) was performed using ImageJ with “aNMJ-morph” macro and BinaryConnectivity plugin, which were described previously (Minty et al., 2020). 20 individual pre- and post-synaptic morphological features were analyzed. A total of 196 NMJs were analyzed: 83 NMJs from *n* = 3 *Scn8a*^T767I/+^ mice and 113 NMJs from n = 3 *Scn8a*^+/+^ littermates with a range of 18–48 NMJs analyzed per muscle from each mouse.

### 2.10. Statistical analysis

Statistical analyses were performed using GraphPad Prism version 10.0 (GraphPad Software, San Diego, CA). One-way ANOVA followed by Tukey’s multiple comparisons test was used to compare brain mRNA levels. Survival data were analyzed with the log-rank (Mantel-Cox) test with two-stage step-up method by Benjamini, Krieger, and Yekutieli for multiple comparisons. Mann-Whitney *U* tests were used for electrophysiology, gait assessment and NMJ morphology where data were not normally distributed and required comparison between two independent groups. Fisher’s exact test was used for distribution of actional potentials, and two-way ANOVA was used to for main effects and interaction of genotype with current-step amplitude in current-frequency data. Body weight data were analyzed using multiple unpaired *t*-tests with Welch correction. Kruskal-Wallis test with Dunn’s post hoc test (nonparametric) or Brown-Forsythe and Welch ANOVA with Dunnett’s T3 post hoc test (parametric) was used to compare behaviors of mouse cohorts during motor phenotyping.

## 3. Results

### 3.1. Generation of the inducible Scn8a-T767I allele in mouse

**Fig. 1.**
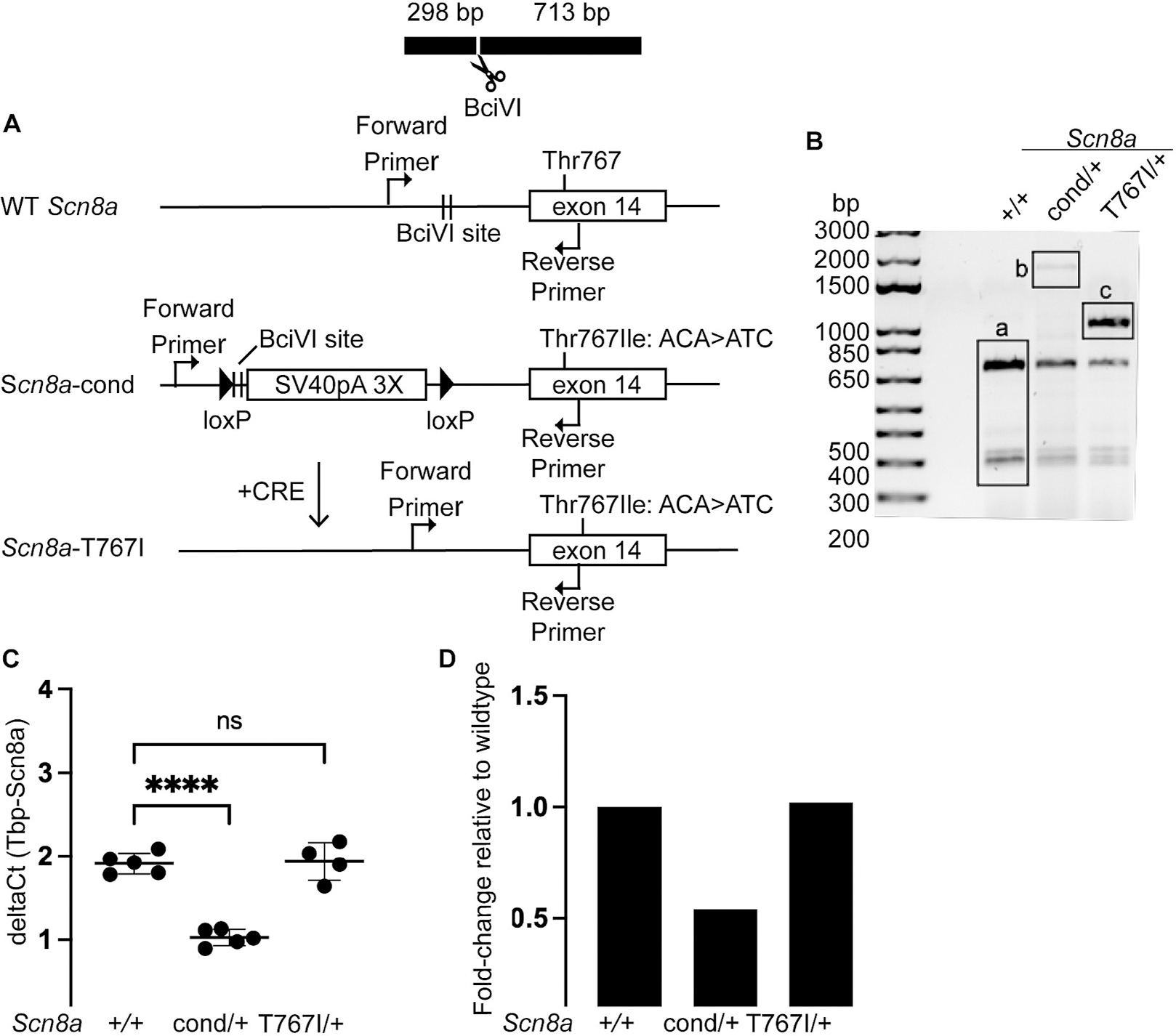
Structure and expression of the conditional *Scn8a*-T767I allele. (A) Residue Thr767 (Thr765 in mouse) is located in exon 14 of the *Scn8a* gene in wildtype mice (top). The lox-stop-lox SV40 polyA 3× termination signal was inserted upstream of exon 14, and the missense mutation Thr767Ile (T767I, nucleotide change ACA > ATC) was knocked into exon 14 in *cis* to the lox-stop-lox sequence, generating the conditional *Scn8a*-cond allele (middle). CRE recombinase removes the stop signal, resulting in expression of the T767I allele in *Cre*-expressing cells (bottom). **(B)** PCR followed by *Bci*VI digestion discriminates between wildtype (+/+), conditional (cond/+), and *Sox2-cre*-mediated activation of T767I (T767I/+) alleles of *Scn8a.* Positions of primers and BciVI restriction site are shown in 1 A. **(C)** Brain mRNA was isolated from *Scn8a*^+/+^, *Scn8a*^cond/+^, and *Scn8a*^T767I/+^ mice. Biological replicates (dots) showed excellent concordance. The deltaCt values indicate the relative abundance of *Scn8a* transcripts corrected for amplification of the internal control, *Tbp*. * = *p* < 0.05, data were analyzed by one-way ANOVA followed by Tukey’s multiple comparisons test. **(D)** *Scn8a*^cond/+^ mice had a 50% reduction in expression of *Scn8a* mRNA compared to *Scn8a*^+/+^ mice. *Sox2-cre* induced expression of the T767I mutation and restored total *Scn8a* mRNA expression to wildtype level.

The *SCN8A*-T767I variant has been identified in at least three unrelated individuals (Encinas et al., 2020; Estacion et al., 2014; Gardella et al., 2018; Johannesen et al., 2018). Individuals heterozygous for the T767I variant have myoclonic jerks and stiffness in the neonatal period followed by onset of treatment-resistant seizures before 2 months of age. Progressive motor impairment was observed in all individuals with the T767I variant, ranging from dyskinesia to profound hypotonia with spasticity in the extremities and loss of ambulation. We predicted that a mouse model carrying the *Scn8a*-T767I variant would display motor phenotypes, which would enable investigation of the mechanisms underlying motor impairment in *SCN8A* DEE. Residue Thr767 is located in exon 14 of the mouse *Scn8a* gene. We generated a conditional knock-in mouse carrying the *Scn8a*-T767I allele utilizing a flox-stop strategy (Dragatsis and Zeitlin, 2001) to generate an inducible allele (Fig. 1A). CRISPR/Cas9 was used to knock-in a lox-stop-lox cassette together with the p.Thr767Ile mutation in the C57BL/6J mice. The lox-stop-lox cassette consists of a SV40 polyA transcription termination signal that is flanked by loxP sites. The lox-stop-lox cassette was inserted upstream of exon 14 to inhibit constitutive expression of the *Scn8a*-T767I allele. Exposure to CRE recombinase deletes the SV40 polyA sequence and restores expression of the allele, generating *Scn8a*^T767I/+^ heterozygosity in any *Cre*-expressing cells. Correct targeting was verified by long-range PCR and sequencing (Suppl Fig. 1A). Targeted mice are herein referred to as *Scn8a*^cond/+^ mice.

*Scn8a*^cond/+^ mice were backcrossed to C57BL/6J mice for several generations to eliminate any off-target effects of the CRISPR targeting. We crossed *Scn8a*^cond/+^ mice with a *Sox2-cre* mouse line (Jackson Laboratory stock #008454) to generate mice with constitutive, global heterozygous expression of *Scn8a*-T767I. *Sox2-cre* is expressed in epiblast cells at embryonic day 6.5 and is active in the female germline(Hayashi et al., 2002, p. 2), thus all of the resulting offspring carrying the *Scn8a*-cond allele will be converted to the *Scn8a*^T767I/+^ genotype. A genotyping assay that takes advantage of differential digestion by the *Bci*VI enzyme permits identification of mice/tissues that have undergone CRE-mediated recombination to generate the activated *Scn8a*-T767I allele (Fig. 1B).

To verify that *Sox2-cre* expression efficiently induced the *Scn8a*-T767I allele, we measured levels of *Scn8a* mRNA in *Scn8a*^+/+^, *Scn8a*^cond/+^, and *Scn8a*^T767I/+^, *Sox2-cre*+ mouse brains using a previously validated Taqman assay for total mouse *Scn8a* expression (Bunton-Stasyshyn et al., 2019). The *Scn8a*^cond/+^ mice had a 50% reduction in *Scn8a* expression compared to *Scn8a*^+/+^ littermates, as expected due to the lox-stop-lox sequence in the conditional allele (Fig. 1C–D). As predicted, *Sox2-cre* restored total *Scn8a* expression similar to wildtype levels in Scn8a^T767I/+^mice due to removal of the SV40 polyA sequence that permits complete restoration of expression from the conditional allele (Fig. 1C–D).

### 3.2. Scn8a^T767I/+^, Sox2-cre + mice exhibit seizures and premature lethality

**Fig. 2.**
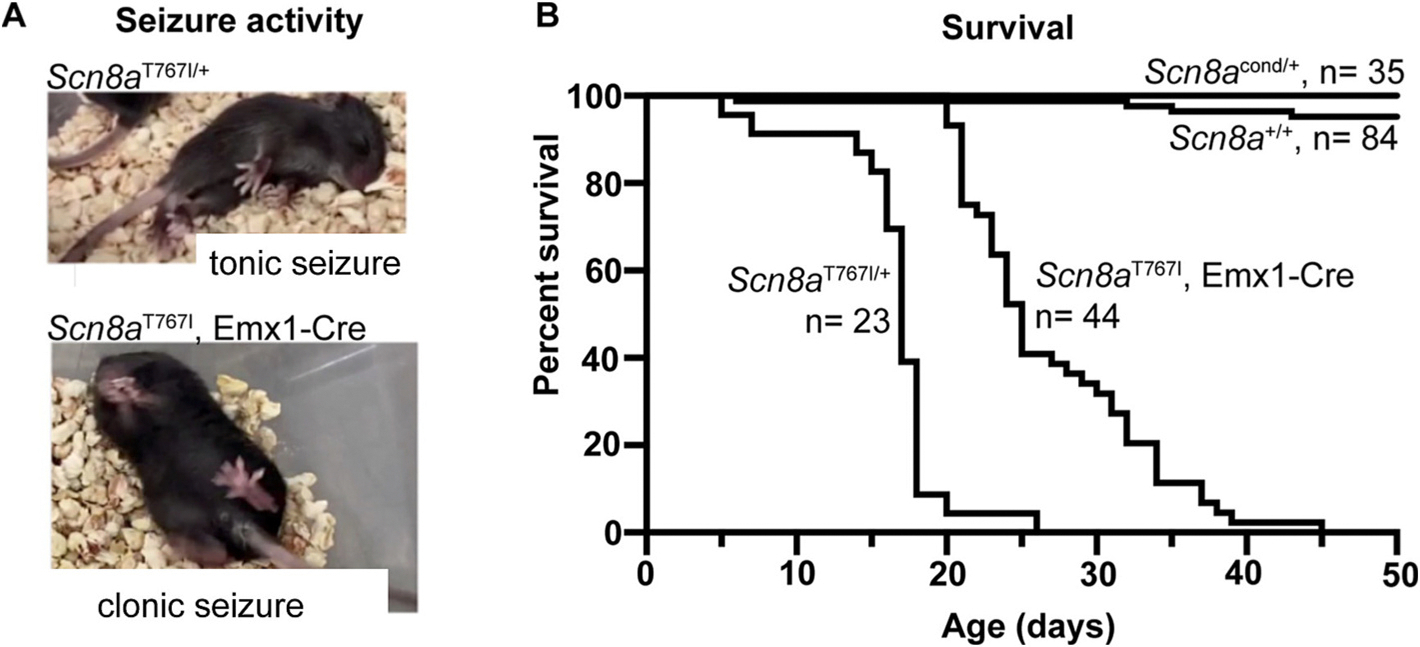
*Scn8a*^T767I^ mutant mice have seizures and premature lethality. (A) *Scn8a*^T767I/+^ (T76I, Sox2-Cre) mice have seizure onset as early as PND 13. Mice with expression of *Scn8a*-T767I in forebrain excitatory neurons (T767I, Emx1-Cre) also exhibit seizures. **(B)** *Scn8a*^T767I/+^ mice (*n* = 44) die prior to weaning age of PND 21 and have a median survival age of PND 17. *Scn8a*^T767^, Emx1-Cre mice (*n* = 25) also exhibit juvenile lethality with a median survival age of PND 25. *Scn8a*^+/+^ mice and *Scn8a*^cond/+^ littermates did not have seizures or seizure-associated premature lethality. The survival curves of *Scn8a*^*T767I/*+^ and *Scn8a*^*T767I*^, Emx1-Cre mice were significantly different (*p* < 0.0001) from *Scn8a*^+/+^. Survival data were analyzed by log-rank (Mantel-Cox) test with multiple comparisons using the two-stage step-up method by Benjamini, Krieger, and Yekuitieli.

Mouse pups were monitored daily starting at PND 5 for seizure activity. Heterozygous *Scn8a*^T767I/+^, *Sox2-cre*+ mice develop generalized tonic-clonic seizures as early as post-natal day (PND) 13 (Fig. 2A). *Scn8a*^T767I/+^ mice typically die within 48 h of seizure onset following a fatal seizure ending in tonic hindlimb extension, with the majority dying prior to weaning age of PND 21 (Fig. 2B). Expression of the *Scn8a*-R1872W allele in forebrain excitatory neurons was previously shown to be sufficient for generation of seizures in mice (Bunton-Stasyshyn et al., 2019). We found that expression of the T767I allele in forebrain-specific excitatory neurons using Emx1-Cre and was also sufficient to induce seizures, usually between PND 13 to PND 15. Seizures in *Scn8a*^T767I^, Emx1-Cre+ mice started as limbic seizures with secondary generalization. *Scn8a*^T767I^, Emx1-Cre+ mice had a slightly extended survival with premature death occurring at a median age of PND 25 (Fig. 2B). These data indicate that there is a conserved mechanism of seizure pathogenesis with a prominent role of excitatory neurons in *SCN8A* DEE.

### 3.3. Pyramidal neurons in CA1 hippocampus of Scn8a^T767I/+^ mice are hyperexcitable

**Fig. 3.**
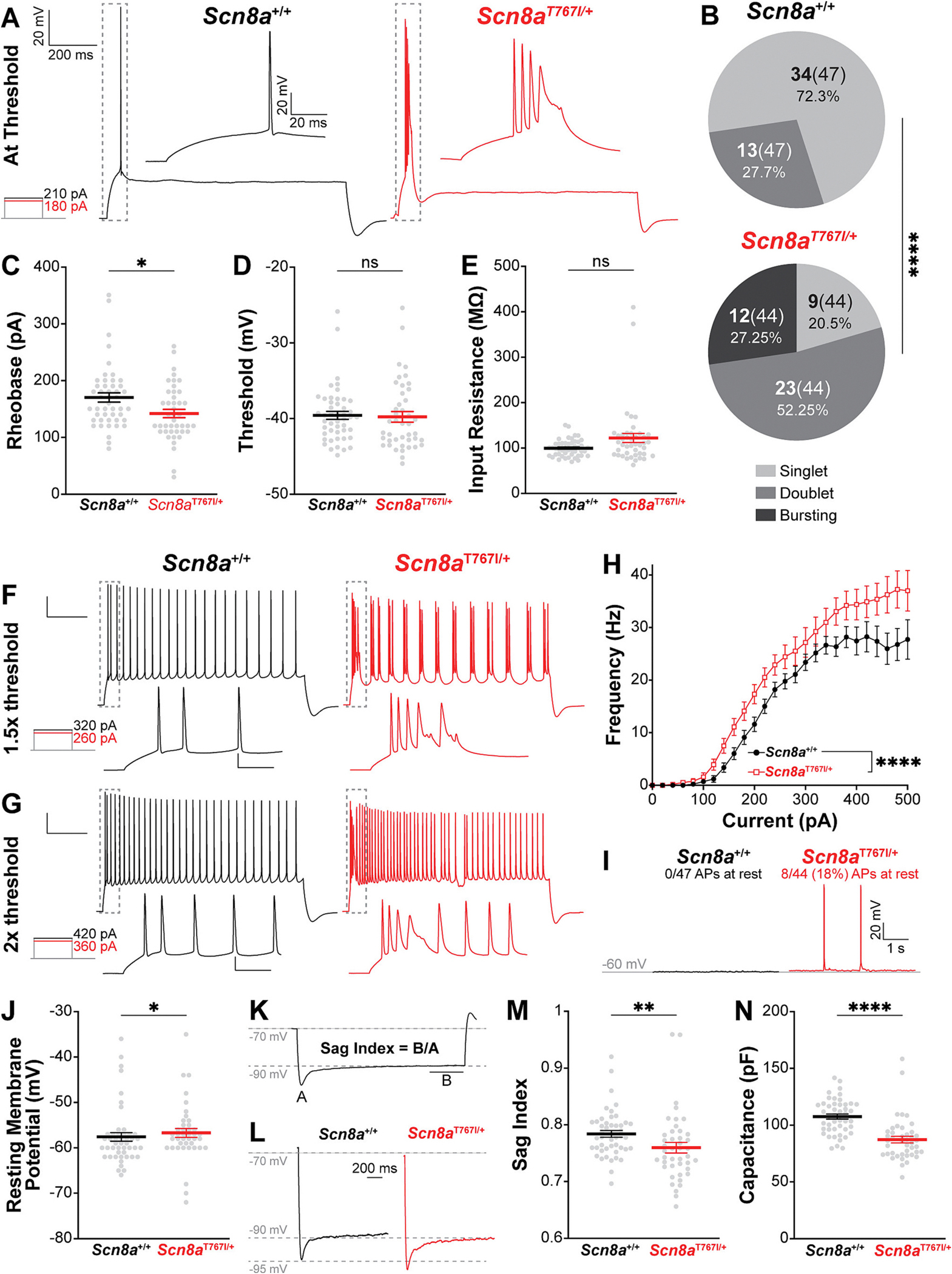
*Scn8a*^*T767I/*+^ mice exhibit hyperexcitability of CA1 hippocampus pyramidal cells. (A) Representative traces of action potentials at threshold. Note a single action potential in *Scn8a*^+/+^ pyramidal cell compared to a burst of action potentials in *Scn8a*^*T767I/*+^ pyramidal cell. All scale bars in figure same as (A) unless otherwise labelled. **(B)** Distribution of action potential characteristics at threshold (singlet, doublet, or bursting) significantly differs between *Scn8a*^+/+^ and *Scn8a*^*T767I/*+^ pyramidal cells, Fisher’s exact test (**** p < 0.0001). **(C)** *Scn8a*^*T767I/*+^ decreases rheobase current, Mann-Whitney *U* test (U = 690.5, ** *p* = 0.0035). Throughout this figure, *Scn8a*^+/+^ data are from 47 pyramidal cells (2 mice) and *Scn8a*^*T767I/*+^ data are from 45 pyramidal cells (2 mice) unless otherwise noted. **(D)** *Scn8a*^*T767I/*+^ does not alter membrane potential threshold for action potential generation, Mann-Whitney U test (U = 934, *p* = 0.3374). **(E)** *Scn8a*^*T767I/*+^ increases input resistance, Mann-Whitney U test (U = 743, * *p* = 0.0137). **(F)** Representative traces of action potentials at 1.5× threshold. Note bursting of every action potential in *Scn8a*^*T767I/*+^ pyramidal cell. **(G)** Distribution of action potential characteristics at 1.5× threshold (singlet, doublet, or bursting) significantly differs between *Scn8a*^+/+^ and *Scn8a*^*T767I/*+^ pyramidal cells, Fisher’s exact test (**** p < 0.0001). Colors same as (B). **(H)** Current-frequency plot shows increased excitability of *Scn8a*^T767I/+^ pyramidal cell relative to *Scn8a*^+/+^, two-way ANOVA main effects of genotype (F[1, 1640] = 100.0, **** p < 0.0001), current step amplitude (F[25, 1640] = 141.0, p < 0.0001), and interaction (F[25, 1640] = 1.857, *p* = 0.0063). **(I)** *Scn8a*^*T767I/*+^ caused 8 of 45 pyramidal cells to fire action potentials at rest. No *Scn8a*^+/+^ pyramidal cells fired action potentials at rest. **(J)** *Scn8a*^*T767I/*+^ depolarizes resting membrane potential, Mann-Whitney U test (U = 767.5, * *p* = 0.0482). *n* = 43 *Scn8a*^*T767I/*+^ pyramidal cells. **(K)** Depiction of voltage sag index calculation. **(L)** Representative traces of voltage sag in *Scn8a*^+/+^ and *Scn8a*^*T767I/*+^ pyramidal cells. Note larger voltage sag of *Scn8a*^*T767I/*+^ pyramidal cell. **(M)** *Scn8a*^*T767I/*+^ increases voltage sag, Mann-Whitney U test (U = 741, * *p* = 0.0131). **(N)** *Scn8a*^*T767I/*+^ decreases capacitance, Mann-Whitney U test (U = 363, **** p < 0.0001). *n* = 46 *Scn8a*^+/+^ pyramidal cells.

Previous work in multiple mouse models found that *Scn8a* GOF variants cause CA1 pyramidal cell hyperexcitability (Bunton-Stasyshyn et al., 2019; Estacion et al., 2014; Lopez-Santiago et al., 2017). To characterize the excitability of CA1 pyramidal cells in *Scn8a*^T767I/+^ mice, we recorded cells in hippocampal slices in current clamp, biased their resting membrane potential to −70 mV, and injected constant amplitude current pulses in steps of 20 pA. At action potential threshold, pyramidal cells in *Scn8a*^T767I/+^ mice were hyperexcitable compared to *Scn8a*^+/+^ mice (Fig. 3A). In *Scn8a*^+/+^ mice, most cells fired a single action potential at threshold (40/47), while in *Scn8a*^T767I/+^ mice the majority (27/45) fired at least two action potentials and some fired bursts of 3 or more (Fig. 3A–B). In *Scn8a*^T767I/+^ mice, the rheobase current for action potential initiation was significantly lower than in *Scn8a*^+/+^ (Fig. 3C), but the voltage threshold was not different (Fig. 3D). This was likely due to a significant increase in input resistance observed in *Scn8a*^T767I/+^ mice (Fig. 3E). Interestingly, this is consistent with a previous *Scn8a*^R1872W/+^ mouse model in which CA1 pyramidal cells also demonstrated lower rheobase current, comparable action potential voltage threshold, and increased input resistance relative to *Scn8a*^+/+^ mice(Bunton-Stasyshyn et al., 2019).

At 1.5 times the rheobase current amplitude, cells in both *Scn8a*^+/+^ and *Scn8a*^T767I/+^ mice fired action potentials throughout the step pulse (Fig. 3F). At this current amplitude, we observed an increase in the number of cells in *Scn8a*^+/+^ mice that fired initial action potential doublets (Fig. 3F, left). However, these cells remained in the minority (13/47), and none fired bursts of three or more action potentials (Fig. 3G). In contrast, most cells in *Scn8a*^T767I/+^ mice fired at least two action potentials (36/45), and many fired ectopic bursts of three or more (13/45) (Fig. 3F–G). Furthermore, at each current step, cells in *Scn8a*^T767I/+^ mice fired more action potentials than those in controls (Fig. 3H). Finally, prior to biasing the resting potential to −70 mV, we observed spontaneous action potentials in *Scn8a*^T767I/+^ mice (8/45 cells) but not in *Scn8a*^+/+^ mice (0/47) (Fig. 3I). These data show that CA1 pyramidal cells are hyperexcitable in *Scn8a*^T767I/+^ mice and are broadly consistent with findings from previous studies in other *Scn8a* gain of function models(Bunton-Stasyshyn et al., 2019; Estacion et al., 2014; Lopez-Santiago et al., 2017).

Finally, we examined subthreshold membrane properties of CA1 pyramidal cells in *Scn8a*^T767I/+^ mice. Prior to biasing the membrane potential to −70 mV, CA1 pyramidal cells in *Scn8a*^T767I/+^ mice had a depolarized resting potential relative to *Scn8a*^+/+^ mice (Fig. 3J). Thus, we next measured membrane voltage sag to assay H-current, which can influence resting membrane potential(Pape, 1996) (Fig. 3K). We found that cells in *Scn8a*^T767I/+^ mice had significantly greater voltage sag (Fig. 3L–M), indicating larger H-current and greater expression of HCN channels. The increase in H-current likely explains the depolarized resting potential in mutant cells(Pape, 1996) (Fig. 3J), but it is at odds with the higher input resistance we observed (Fig. 3E). Thus, other membrane properties may be affected in *Scn8a*^T767I/+^ mice, such as reduced leak current. Finally, we calculated estimated membrane capacitance from membrane time constant and resistance measurements in response to 10 pA current pulses. Strikingly, cells in *Scn8a*^T767I/+^ mice had a significantly lower membrane capacitance (Fig. 3N), suggesting changes in membrane surface area, which could be related to either dendritic branching or spine density.

### 3.4. Scn8a^T767I/+^ mice exhibit motor impairment and muscle weakness

**Fig. 4.**
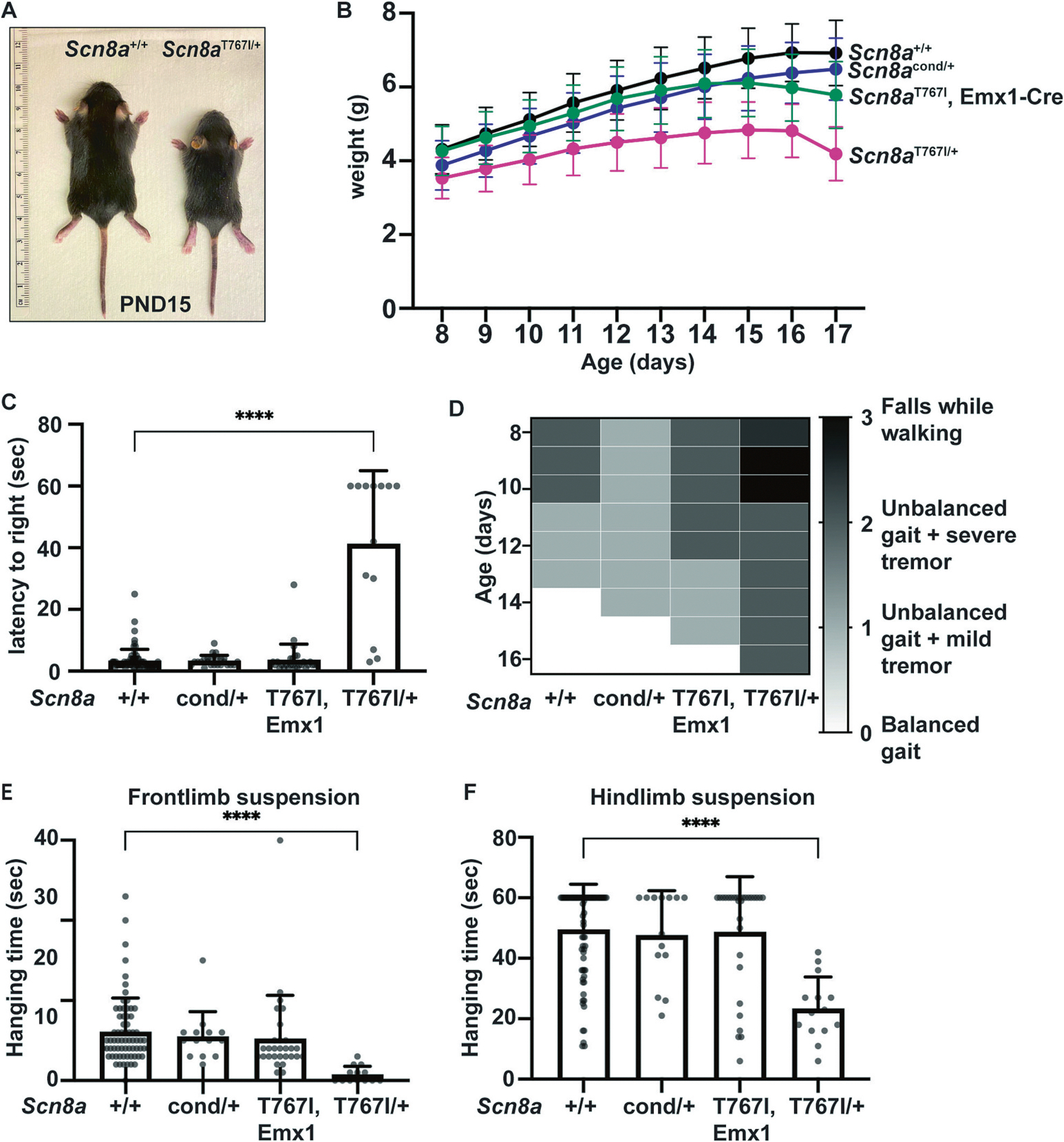
Motor impairment and developmental delay in *Scn8a*^T767I/+^ mice. (A) *Scn8a*^T767I/+^ mice are smaller than age- and sex-matched *Scn8a*^+/+^ littermates. **(B)** *Scn8a*^T767I/+^ mice (n = 25, pink line) show significantly lower body weights (*p* < 0.001) compared to *Scn8a*^+/+^ littermates (*n* = 102, black line) from at all time points from PND 8–17. *Scn8a*^T767I^, Emx1-Cre+ mice (n = 43, green line) did not differ in body weights compared to *Scn8a*^+/+^ mice until after seizure onset (p < 0.001 for PND 15–17 only). There was no difference in body weights between *Scn8a*^cond/+^ mice (n = 25, purple line) and *Scn8a*^+/+^ mice. Data points and error bars represent mean body weights and standard deviation (SD).Differences between groups were analyzed using multiple unpaired *t*-tests with Welch corrections along with multiple comparisons using the two-stage step-up method by Bejamini, Krieger and Yekutieli. **(C)** *Scn8a*^T767I/+^ mice (*n* = 13) showed significantly higher latency to right compared to *Scn8a*^+/+^ mice (*n* = 73). *Scn8a*^cond/+^ (n = 25) and *Scn8a*^T767I^, Emx1-Cre+ (*n* = 29) mice did not differ in latency to right compared to *Scn8a*^+/+^ mice. Histograms represent mean and SD, and groups were analyzed by Kruskal-Wallis test, which showed a significant effect of genotype on righting time, *H* ([df = 3], [*N* = 140]) = 33.10, *p* < 0.0001. Post-hoc comparisons using Dunn’s test showed that median righting time was significantly higher for *Scn8a*^T767I/+^ compared to *Scn8a*^+/+^ (p < 0.0001). **(D)** Heatmap in grey-scale showing median gait scores for mice from PND 8–16. White cells (score 0) represent a balanced gait without tremor, light grey cells (score 1) represent walking with slightly unbalanced gait with mild tremor, dark grey cells (score 2) represent walking with unbalanced gait with severe tremor, and black cells (score 3) represent mice falling while walking. *Scn8a*^T767I/+^ mice (*n* = 16) show severe unbalanced gait with falls on PND 9 and PND 10. *Scn8a*^T767I/+^ mice have significantly worse gait scores compared to the other three groups (*n* > 14 per group) which develop balanced gait by PND 16. Gait severity between groups was analyzed by Mann-Whitney test with two-stage step-up method by Benjamini, Krieger, and Yekutieli. *Scn8a*^T767I/+^ mice differed from *Scn8a*^+/+^ mice at each timepoint tested (*p* < 0.00001). *Scn8a*^cond/+^ and Scn8aT767I, Emx1-Cre mice did not differ from *Scn8a*^+/+^ mice. **(*E*-F)** *Scn8a*^T767I/+^ mice show significantly (p < 0.0001) less hanging time in both frontlimb suspension test (PND 10, *n* > 13 per group) and hindlimb suspension test (PND 8, n > 14 per group) compared to the other three groups. Histograms represent mean and SD. Frontlimb data were analyzed by Kruskal-Wallis test (*H* [3, 125] = 34.36, p < 0.0001). Hindlimb data were analyzed by Brown-Forsythe (F* [DFn, DFd] = 12.98 [3, 68.36], p < 0.0001, *N* = 132) and Welch (W [DFn, DFd] = 21.79 [3, 36.00], p < 0.0001, N = 132) ANOVA. Post-hoc tests for frontlimb (Dunn’s) and hindlimb (Dunnett’s T3) data showed that median suspension time was significantly lower for *Scn8a*^T767I/+^ compared to *Scn8a*^+/+^ (p < 0.0001). (For interpretation of the references to colour in this figure legend, the reader is referred to the web version of this article.)

Heterozygous *Scn8a*^T767I/+^, Sox2-Cre+ mice are smaller than their *Scn8a*^+/+^ and *Scn8a*^cond/+^ littermates (Fig. 4A). *Scn8a*^T767I/+^ mice weigh significantly less than littermates at all time points (Fig. 4B). In contrast, *Scn8a*^T767I^, Emx-Cre+ mice gain weight normally until seizure onset (~PND15). Thus, the reduction in weight requires expression of the T767I allele in cell types other than forebrain excitatory neurons. To examine motor development prior to seizure onset in *Scn8a*^T767I/+^, Sox2-Cre+ mice, we performed the righting reflex test on mice aged PND8–10. Wildtype mice that are placed in a prone position rapidly turn themselves over into an upright position (Fig. 4C). *Scn8a*^cond/+^ and *Scn8a*^T767I^, Emx1-Cre+ mice demonstrated no change in righting ability. However, *Scn8a*^T767I/+^, Sox2-Cre+ mice had significantly reduced ability to right, with some mice unable to right themselves at all during the 60-s trial (Fig. 4C, Suppl Fig. 2A). These data demonstrate that *Scn8a*^T767I/+^, Sox2-Cre+ mice exhibit altered motor development.

We also performed gait analysis on mice aged PND8–16 that had not exhibited seizures. Gait scores were assigned to mice by observing their ambulatory behavior on a flat surface. The median gait score of *Scn8a*^+/+^ mice shows that mice typically progress in gait efficiency from slightly unbalanced gait on PND8 to balanced gait on PND16 (Fig. 4D). *Scn8a*^cond/+^ and *Scn8a*^T767I^, Emx1-Cre+ mice also show this trajectory. In contrast, the median gait score of *Scn8a*^T767I/+^, Sox2-Cre+ mice demonstrates an unbalanced gait with severe tremors and more falls on PND 9 and PND 10 compared to *Scn8a*^+/+^ littermates (Fig. 4D). *Scn8a*^T767I/+^ mice never achieve balance gait. These data suggest that *Scn8a*^T767I/+^, Sox2-Cre+ mice exhibit persistent motor impairment prior to seizure onset.

Due to their reduced mass and motor dysfunction, we hypothesized that muscle strength would be impaired in *Scn8a*^T767I/+^, Sox2-Cre+ mice. We tested forelimb and hindlimb strength using limb-hanging tests developed for very young mice(Feather-Schussler and Ferguson, 2016) and measured the latency of the mice to fall. The *Scn8a*^T767I/+^ mice spent significantly less time hanging from either frontlimbs or hindlimbs compared to *Scn8a*^+/+^ littermates (Fig. 4E–F). Frontlimb and hindlimb weakness persists in *Scn8a*^T767I/+^, Sox2-Cre+ mice during the timepoints measured between PND 8 and PND 14 (Suppl Fig. 2B-C). *Scn8a*^cond/+^ and *Scn8a*^T767I^, Emx1-Cre+ mice did not exhibit impaired limb strength. There were no significant differences between *Scn8a*^+/+^, Sox2-Cre- and *Scn8a*^+/+^, Sox2-Cre+ mice in survival, body weight, or for any of the motor tests, indicating that expression of Sox2-Cre does not contribute to abnormal phenotypes in *Scn8a*^T767I/+^ mice. Taken together, these data suggest that *Scn8a*^T767I/+^ mice exhibit significant, early onset motor impairment. In contrast to seizure pathogenesis, expression of the T767I allele in excitatory neurons alone is not sufficient to generate motor impairment. Thus, these data indicate that the neuronal mechanisms underlying motor impairment are distinct from those underlying seizures.

### 3.5. Scn8a^T767I/+^ mice demonstrate impaired motor neuron function and reduced number of motor units

**Fig. 5.**
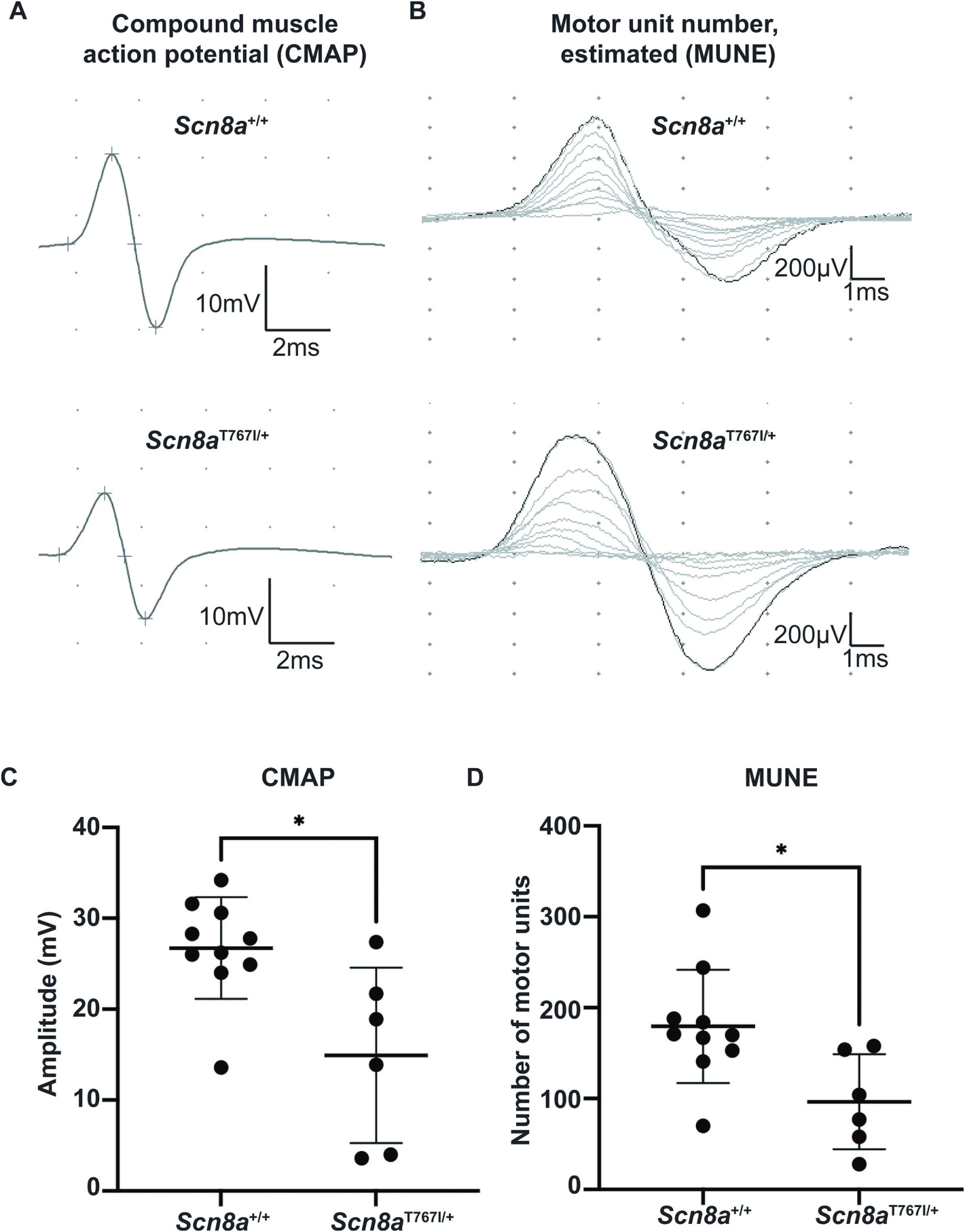
Motor unit dysfunction in *Scn8a*^T767I/+^ mice. (A) Compound muscle action potential (CMAP) amplitude was recorded from right triceps surae after sciatic nerve stimulation. Representative CMAP traces shown for *Scn8a*^+/+^ and *Scn8a*^T767I/+^ mice. **(B)** Representative traces for motor unit number estimation (MUNE) shown for *Scn8a*^+/+^ and *Scn8a*^T767I/+^ mice. **(C)** *Scn8a*^T767I/+^ mice show significantly reduced CMAP amplitude compared to *Scn8a*^+/+^ mice. Data points represent CMAP amplitude of each mouse. **(D)** MUNE indicates significantly fewer functional motor units in *Scn8a*^T767I/+^ mice compared to *Scn8a*^+/+^ mice. Data points represent MUNE estimate of each mouse. Histograms represent mean and SD, respectively. Mann-Whitney test was performed to confirm the statistical difference (*p* < 0.02) between groups.

We hypothesized that motor impairment in *Scn8a*^T767I/+^ mice could be driven in part by dysfunction of motor neurons innervating skeletal muscle. We tested motor unit function by performing electrophysiological measurements in the sciatic nerve. We measured compound muscle action potential (CMAP) from the right triceps surae in *Scn8a*^T767I/+^ mice at PND11. We found that CMAP, which is the total electrophysiological output of the muscle obtained by applying supramaximal stimulation to the nerve, was reduced in *Scn8a*^T767I/+^ mice (Fig. 5A, C). We also measured single unit motor potential (SMUP) by incrementally stimulating the sciatic nerve to individually recruit and record electrical potentials from single motor units. A motor unit number estimation (MUNE) was then calculated by dividing the maximum CMAP amplitude (peak-to-peak) by the average SMUP amplitude (peak-to-peak). The MUNE represents the electrophysiologic estimation of the number of motor units in a particular muscle. We found that MUNE was reduced in *Scn8a*^T767I/+^ mice (Fig. 5B, D), indicating that there are fewer functional motor units. These data suggest that motor neuron function is disrupted in *SCN8A* DEE.

### 3.6. Scn8a^T767I/+^ mice have altered neuromuscular junction morphology

**Fig. 6.**
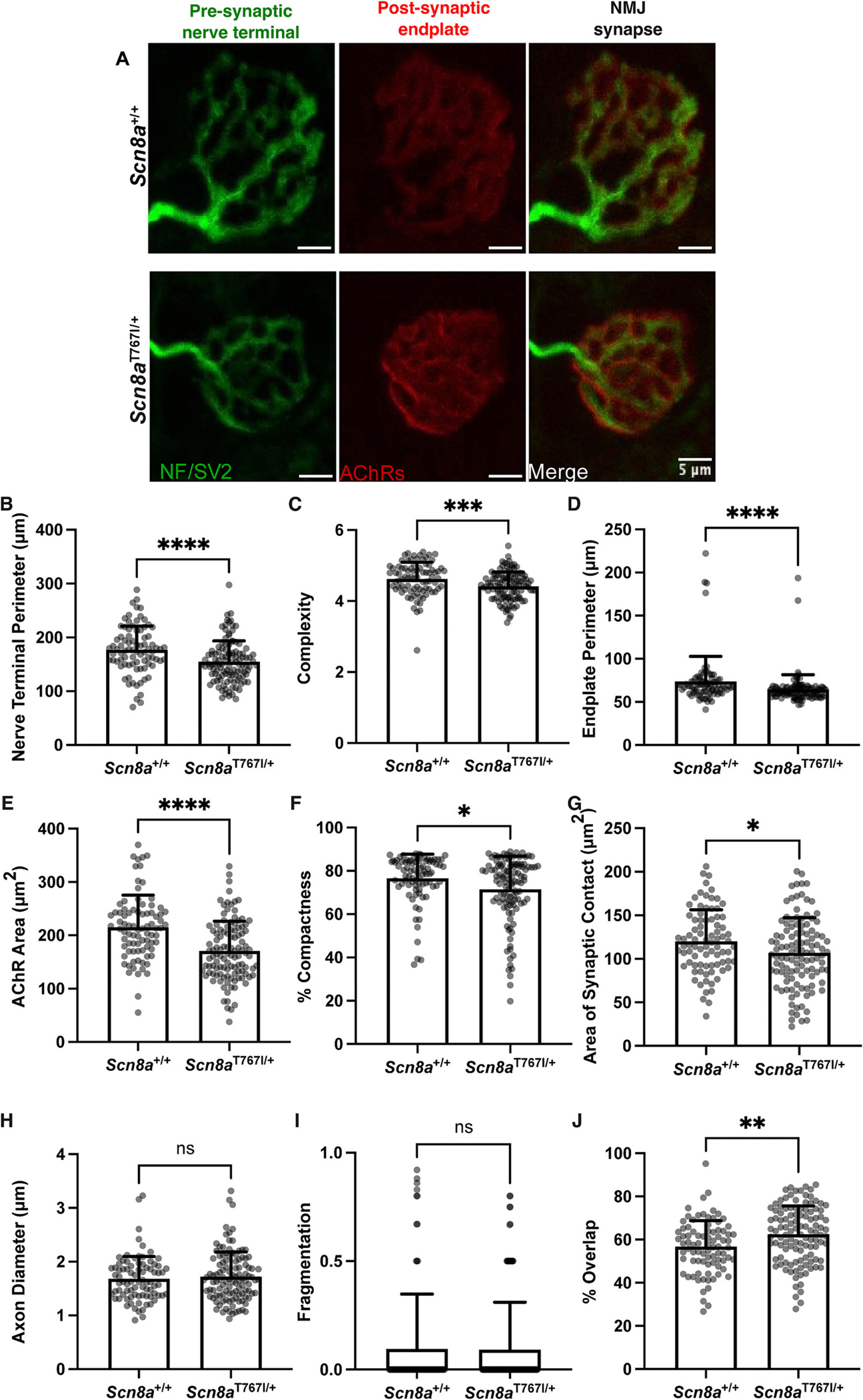
Altered NMJ morphology in *Scn8a*^T767I/+^ mice. (A) Immunofluorescence images were collected of whole-mount gastrocnemius muscle fibers of *Scn8a*^T767I/+^ mice and littermate *Scn8a*^+/+^ mice at age PND 15. Green shows presynaptic nerve terminals labelled with anti-2H3/SV2 antibodies. Red shows postsynaptic endplate acetylcholine receptors (AChRs) on muscle fibers stained with α-bungarotoxin. **(B)** Size of presynaptic nerve terminals is reduced in *Scn8a*^T767I/+^ NMJs compared to *Scn8a*^+/+^ NMJs (149.9 vs 176.4, p < 0.0001). **(C)** Branching complexity is reduced in *Scn8a*^T767I/+^ NMJs (4.4 vs 4.7, (*p* = 0.0003). **(D)** Postsynaptic endplates are smaller in *Scn8a*^T767I/+^ NMJs (62.0 vs 68.1, p < 0.0001). **(E)** Area encompassed by AChRs is decreased in *Scn8a*^T767I/+^ NMJs (158.7 vs 200.3, *p* < 0.0002). **(F)** NMJ compactness is reduced in *Scn8a*^T767I/+^ mice (75.9 vs 79.7, *p* = 0.02). **(G)** Synaptic contact is reduced in *Scn8a*^T767I/+^ NMJs (75.9 vs 79.7, p = 0.02). **(H)** Diameter of axons innervating NMJs is not different between *Scn8a*^T767I/+^ and *Scn8a*^+/+^ mice. **(I)** Fragmentation of NMJs is not different between *Scn8a*^T767I/+^ and *Scn8a*^+/+^ mice. **(J)** *Scn8a*^T767I/+^ NMJs have higher percent overlap (63.9 vs 57.5, *p* = 0.001) but are innervated similarly to *Scn8a*^+/+^ mice and do not show signs of denervation or degeneration. Histograms represent group mean and SD, respectively. Each dot represents one NMJ from *Scn8a*^T767I/+^ (83 NMJs analyzed from *n* = 3 mice) or *Scn8a*^+/+^ littermates (113 NMJs analyzed from n = 3 mice). Difference between groups was tested using Mann-Whitney test. (For interpretation of the references to colour in this figure legend, the reader is referred to the web version of this article.)

To investigate connectivity between the motor neuron and muscle in *Scn8a*^T767I/+^ mice, we analyzed neuromuscular junction (NMJ) morphology in gastrocnemius muscle at PND 15 (Fig. 6A). A total of 20 parameters were evaluated (Suppl. Table 1). We identified significant morphological alterations in NMJs from *Scn8a*^T767I/+^ mice. The perimeters of presynaptic nerve terminals were smaller and branching complexity was reduced in *Scn8a*^T767I/+^ mice compared to *Scn8a*^+/+^ littermates (Fig. 6B–C). Postsynaptic endplates were also smaller in *Scn8a*^T767I/+^ mice (Fig. 6D). Acetylcholine receptor (AChR) area and NMJ compactness, a measure of AChR dispersal, were reduced in *Scn8a*^T767I/+^ mice (Fig. 6E–F). We also observed reduced synaptic contact in NMJs of *Scn8a*^T767I/+^ mice (Fig. 6G). These data show that *Scn8a*^T767I/+^ mice exhibit significant NMJ dysmorphology and suggest that the functional capacity of the NMJs is reduced.

The reduction in size and branching complexity of the NMJ suggests that NMJ maturation is delayed in *Scn8a*^T767I/+^ compared to *Scn8a*^+/+^ mice. However, the majority of NMJs in *Scn8a*^T767I/+^ mice were mono-innervated, indicating that maturation of the NMJ was not arrested completely. We did not detect any differences in axon diameter between *Scn8a*^T767I/+^ and *Scn8a*^+/+^ mice at innervated NMJs (Fig. 6H). We measured fragmentation and occupancy (% overlap) of the NMJs to assess whether there were any signs of degeneration. We found no difference in fragmentation between *Scn8a*^T767I/+^ and *Scn8a*^+/+^ NMJs (Fig. 6I). We observed slightly higher overlap in *Scn8a*^T767I/+^ mice, likely due to the smaller size of the NMJ, indicating that the NMJs are not degenerating at PND 15 (Fig. 6J). Morphological changes in NMJs may precede denervation and degeneration, but we are unable to assess later timepoints because of the early lethality of *Scn8a*^T767I/+^ mice. Overall, these data suggest that morphological abnormalities of the NMJ may reduce synaptic function and lead to unreliable neuromuscular transmission in *SCN8A* DEE.

## 4. Discussion

Alterations in Na_v_1.6 channel activity are associated with motor dysfunction in humans. Motor impairment, muscle weakness, and movement disorders including ataxia, dystonia, dyskinesia, and choreoathetosis are seen in *SCN8A* DEE, which is caused primarily by GOF variants in *SCN8A*. Reduced Na_v_1.6 function has been identified in individuals with movement disorders without convulsive seizures (Gardella and Møller, 2019; Wagnon et al., 2017, 2018). A recent large study of missense variants of *SCN8A* identified in episodic or chronic ataxia found that strong LOF effects were associated with chronic ataxia and milder LOF effects were associated with episodic ataxia (Lyu et al., 2023).

Mice with complete loss of Na_v_1.6 exhibit reduced motor nerve conduction velocity, widening of the non-myelinated gaps at the nodes of Ranvier, failure and degeneration of the neuromuscular junction (NMJ), and morphological changes in the muscle that are consistent with functional denervation, including terminal sprouting of the motor nerves and muscle fiber atrophy (Kearney, 2002; Meisler et al., 2001). In contrast, mechanisms underlying motor impairment related to Na_v_1.6 GOF have not been examined because previous mouse models of *SCN8A* DEE did not exhibit motor dysfunction. We developed a new mouse model of *SCN8A* DEE with a conditional GOF allele to investigate motor pathophysiology.

The *SCN8A*-T767I mutation causes premature activation of the channel with no change in steady state inactivation kinetics, resulting in a larger window current (Estacion et al., 2014; Pan and Cummins, 2020). Increases in persistent and resurgent currents were also recorded (Pan and Cummins, 2020). The *Scn8a*^T767I/+^ mouse exhibits the spontaneous seizures and premature lethality characteristic of *SCN8A* DEE. Hippocampal neurons are hyperexcitable in *Scn8a*^T767I/+^ mice, which is consistent with brain slice recordings from the other two existing *SCN8A* DEE mouse models. Thus, we conclude that *Scn8a*^T767I/+^ mice represent a valid new model for *SCN8A* DEE.

### 4.1. Scn8a^T767I/+^ is the first mouse model of SCN8A DEE to exhibit motor impairment

Although the previous mouse models of *SCN8A* DEE exhibited seizures, they did not successfully model the motor comorbidities that are prevalent in the disorder. Using a battery of behavioral tests designed to assess motor function in neonatal mice, we showed that *Scn8a*^T767I/+^ mice have significant motor impairment. *Scn8a*^T767I/+^ mice have impaired righting reflex, reduced muscle strength, and gait abnormalities that do not resolve with age. Thus, our data indicate that *Scn8a*^T767I/+^ mice exhibit profound delays in motor development, significant muscle weakness, and severe motor impairment consistent with the prominent motor comorbidities observed in the majority of *SCN8A* DEE patients. The T767I variant primarily causes premature activation of Na_v_1.6, whereas the previous mouse models had variants (N1768D and R1872W) that primarily cause impaired inactivation of Na_v_1.6. We hypothesize that these different biophysical defects may contribute to the differential expression of motor phenotypes in mice.

The inducible design of the conditional T767I allele allows us to investigate which cell types and neuronal circuits contribute to motor impairment in *SCN8A* DEE.

### 4.2. Distinct neuronal circuits contribute to seizures and motor impairment in Scn8a^T767I^ mice

Our characterization of *Scn8a*^T767I^ mice demonstrates that expression of the T767I in different neuronal populations causes seizures and motor impairment. Scn8a^cond/+^ mice do not differ from *Scn8a*^+/+^ mice in survival, body weight, or motor function. *Scn8a*^cond/+^ mice are equivalent to the *Scn8a*^+/−^ genotype with 50% reduction of *Scn8a*. Our results are consistent with previous studies demonstrating that mice heterozygous for null mutations of *Scn8a* do not exhibit motor impairment (Burgess et al., 1995; McKinney et al., 2008). Mice with forebrain-specific expression of the *Scn8a*-T767I allele develop focal seizures that evolve into generalized seizures leading to premature death within two weeks of onset, but they do not exhibit abnormal motor phenotypes.

We hypothesize that additional neuronal subtypes are involved in generating the full clinical phenotype in *SCN8A* DEE. The corticospinal tract is made up of pyramidal descending neurons of the motor cortex and spinal motor neurons. Neurons from the motor cortex synapse onto spinal motor neurons, which in turn terminate at neuromuscular junctions, to propagate motor signals to the muscles. Because forebrain-specific expression of the T767I allele did not produce motor impairment, we excluded pyramidal neurons of the motor cortex as the primary driver of motor impairment and turned our attention to spinal motor neurons. It is also possible that dysfunction of cerebellar neurons contributes to motor impairment in *Scn8a*^T767I/+^ mice. The conditional allele we have generated will allow us to assess the contributions of additional neuronal subtypes, such as cholinergic motor neurons and cerebellar Purkinje neurons, to motor impairment in future studies.

### 4.3. Motor units are dysfunctional in Scn8a-T767I/+ mice

Motor units are defined as a motor neuron, neuromuscular junction, and the skeletal muscle fibers innervated by the motor neuron. Compound muscle action potential (CMAP) and motor unit number estimation (MUNE) measurements assess the functional status of the motor unit pool and are particularly relevant for movement disorders like those seen in *SCN8A* DEE. CMAP response measures the output of the motor units supplying a particular muscle or group of muscles, and MUNE estimates the number of motor units supplying a particular muscle. We found that both Max CMAP and MUNE were significantly reduced in *Scn8a*^T767I/+^ mice.

We found significant NMJ dysmorphology in *Scn8a*^T767I/+^ mice. We identified smaller presynaptic nerve terminals and postsynaptic motor endplates in *Scn8a*^T767I/+^ mice, along with reductions in branching complexity and synaptic contact. We did not observe any hallmarks of denervation or degeneration of NMJs. Quantal content is proportional to motor nerve terminal size (Harris and Ribchester, 1979). The reduced size NMJs in *Scn8a* mutant mice suggests that the NMJs have reduced functional capacity and are not able to release sufficient neurotransmitter to elicit an action potential in the muscle fiber. Changes in NMJ architecture are an early sign of motor dysfunction and have been observed in peripheral nerve diseases like amyotrophic lateral sclerosis and Charcot-Marie-Tooth diseases. More detailed analyses of NMJ function in *Scn8a*^T767I/+^ mice will be necessary to determine whether failure of neuromuscular transmission is a driver of motor impairment in *SCN8A* DEE.

### 4.4. The effects of SCN8A GOF on motor pathophysiology

The pathological role of GOF *SCN8A* DEE variants has been demonstrated in several brain regions and neuron types, including enhanced excitability of excitatory pyramidal cells in the cerebral cortex and hippocampus (Bunton-Stasyshyn et al., 2019; Lopez-Santiago et al., 2017). However, the effects of pathogenic variants on the function of neurons in motor networks, including spinal motor neurons, has not been reported. Our study is the first report of dysfunctional motor units in a mouse model of *SCN8A* epilepsy. These data suggest that motor neuron function is impaired by the *Scn8a*-T767I variant and that dysfunction of motor neurons may underlie motor impairment and movement disorders in *SCN8A* DEE. A limitation of the current study is that we have not directly recorded from spinal motor neurons. More work is required to identify the underlying molecular and cellular mechanisms by which GOF *SCN8A* DEE variants disrupt motor unit function, including measurement of motor neuron electrophysiology in *Scn8a*^T767I/+^ mice.

Given our finding that hippocampal CA1 neurons in *Scn8a*-T767I/+ mice are hyperexcitable, it is reasonable to predict that motor neurons in *Scn8a*^T767I/+^ mice may also be hyperexcitable. Hyperexcitability of motor neurons and altered sodium channel kinetics have been detected in several motor neuron diseases, including spinal muscular atrophy (SMA), amyotrophic lateral sclerosis (ALS), and some types of Charcot-Marie Tooth disease (Geevasinga et al., 2015; H. Liu et al., 2015; Mogyoros et al., 1998; Quinlan et al., 2019; Saporta et al., 2015; Zona et al., 2006). In SMA, motor neuron hyperexcitability precedes loss of motor units (Quinlan et al., 2019). The use of sodium channel blockers can rescue motor neuron death (Rojas et al., 2014). Taken together, these data suggest that some GOF variants in *SCN8A* may cause motor neuron hyperexcitability that leads to a loss of motor units underlying motor impairment in *SCN8A* DEE.

We observed a reduced number of functional motor units in *Scn8a*-T767I/+ mice, which resembles *Scn8a* loss-of-function. *SCN8A* variants frequently exhibit a mix of GOF and LOF effects on channel activity (Vanoye et al., 2024), which makes it difficult to predict the overall activity profile for neuronal circuits. Indeed, two variants with mixed GOF and LOF effects were identified in episodic ataxia (Lyu et al., 2023). It will be important to evaluate the effects of the *SCN8A* variants in motor neurons to differentiate between GOF and LOF mechanisms in *SCN8A*-related motor impairment. The conditional *Scn8a*-T767I allele can be expressed in specific neuronal populations to investigate the role of GOF variants and to identify neuronal networks that contribute to motor impairment. For example, this mouse model enables future work to study the role of *Scn8a* and the effects of GOF Na_v_1.6 activity in motor neurons for the first time. *Scn8a*^T767I/+^ mice represent a unique new tool to elucidate pathophysiological mechanisms in *SCN8A* DEE.

## Supplementary Material

1

2

3

Supplementary data to this article can be found online at https://doi.org/10.1016/j.nbd.2026.107317.

## Data Availability

Data will be made available on request.
