# Supplementary material for: Distinct neuronal mechanisms for motor impairment and seizures in a novel mouse model of SCN8A epileptic encephalopathy
Source: Neurobiol Dis. Author manuscript; Available in PMC 2026 May 5. (PMC13141817; doi:10.1016/j.nbd.2026.107317)
Supplement: 3 [file NIHMS2158508-supplement-3.docx]

**Supplemental Table 1**

| Morphological Parameter (units) | *Scn8a*^+/+^ Median value | *Scn8a*^T767I/+^ Median value | *Scn8a*^+/+^ 95% CI (Lower-Upper) | *Scn8a*^T767I/+^ 95% CI (Lower-Upper) | Mann Whitney test P value |
| --- | --- | --- | --- | --- | --- |
| Nerve Terminal Perimeter (μm) | 176.4 | 149.9 | 167.5-186.6 | 147.7-162.1 | <0.0001 |
| Complexity | 4.670 | 4.420 | 4.523-4.726 | 4.342-4.491 | 0.0003 |
| Endplate Perimeter (μm) | 68.10 | 62.01 | 67.50-80.16 | 61.19-67.56 | <0.0001 |
| AChR Area (μm^2^) | 213.2 | 165.0 | 202.0-228.3 | 160.3-181.2 | <0.0001 |
| Compactness (%) | 79.72 | 75.92 | 74.21-79.06 | 68.75-74.40 | 0.0194 |
| Average Area of AChR Clusters (μm^2^) | 200.3 | 158.7 | 176.4-210.1 | 144.7-169.6 | 0.0002 |
| Axon Diameter (μm) | 1.625 | 1.670 | 1.591-1.775 | 1.638-1.809 | 0.6102 |
| Fragmentation | 0.000 | 0.000 | 0.04045-0.1506 | 0.05168-0.1330 | 0.7562 |
| Overlap (%) | 57.53 | 63.88 | 54.13-59.40 | 60.12-64.99 | 0.0011 |
| Nerve Terminal Area (μm^2^) | 122.8 | 126.1 | 121.1-137.5 | 120.6-134.8 | 0.9306 |
| Number of Terminal Branches | 25.00 | 20.00 | 23.58-28.58 | 18.88-22.25 | 0.0008 |
| Number of Branch Points | 21.28 | 17.36 | 20.06-24.09 | 17.40-20.44 | 0.0158 |
| Total Length of Branches (μm) | 94.69 | 81.87 | 88.19-99.11 | 80.12-88.89 | 0.0060 |
| Average Length of Branches (μm) | 3.670 | 4.360 | 3.793-4.678 | 4.472-5.363 | 0.0521 |
| AChR Perimeter (μm) | 135.4 | 131.2 | 138.0-168.4 | 135.6-158.5 | 0.5685 |
| Endplate Diameter (μm) | 24.14 | 21.66 | 23.79-26.32 | 21.42-22.79 | <0.0001 |
| Endplate Area (μm^2^) | 274.7 | 234.4 | 265.2-305.5 | 227.0-251.4 | <0.0001 |
| Unoccupied AChR Area (μm^2^) | 89.80 | 57.29 | 85.40-104.6 | 57.94-69.55 | <0.0001 |
| Number of AChR Clusters | 1.000 | 1.000 | 1.148-1.912 | 1.127-1.404 | 0.7527 |
| Average Area of AChR Clusters (μm^2^) | 200.3 | 158.7 | 176.4-210.1 | 144.7-169.6 | 0.0002 |

**Supplemental Table 1. Morphological analysis of neuromuscular junctions (NMJs).**

**Supplemental Table 2**

| Experiment | Fixed effects  (type III) | P value | P value  summary | Statistically  significant  (P < 0.05)? | F (DFn, DFd) | Geisser-Greenhouse's  epsilon |
| --- | --- | --- | --- | --- | --- | --- |
| Righting Reflex | PND | <0.0001 | **** | Yes | F (1.444, 170.3) = 17.01 | 0.7218 |
|  | Genotype | <0.0001 | **** | Yes | F (3, 136) = 168.1 |  |
|  | PND x Genotype | <0.0001 | **** | Yes | F (4.331, 170.3) = 6.227 | 0.7218 |
| Frontlimb Suspension | PND | 0.7770 | ns | No | F (3.557, 425.0) = 0.4130 | 0.8892 |
|  | Genotype | <0.0001 | **** | Yes | F (3, 129) = 14.65 |  |
|  | PND x Genotype | 0.4306 | ns | No | F (10.67, 425.0) = 1.016 | 0.8892 |
| Hindlimb Suspension | PND | <0.0001 | **** | Yes | F (4.419, 511.1) = 16.36 | 0.7365 |
|  | Genotype | <0.0001 | **** | Yes | F (3, 129) = 14.72 |  |
|  | PND x Genotype | <0.0001 | **** | Yes | F (13.26, 511.1) = 5.403 | 0.7365 |

**Supplemental Table 2. Mixed-effects model statistical analysis for behavioral tests presented in Supplemental Figure 2.**

**Supplemental Table 3**

|  | | Experiment | | |
| --- | --- | --- | --- | --- |
|  | | Righting Reflex | Frontlimb Suspension | Hindlimb Suspension |
| Number of columns (Genotype) | | 4 | 4 | 4 |
| Number of rows (PND) | | 3 | 5 | 7 |
| Number of subjects (Mouse) | | 140 | 133 | 133 |
| Number of subjects per group | *Scn8a*^+/+^ | 60-73 | 58-75 | 59-75 |
|  | *Scn8a*^cond/+^ | 25 | 9-14 | 5-14 |
|  | *Scn8a*^T767I/+^ | 11-13 | 13-15 | 14-15 |
|  | *Scn8a*^T767I^, Emx1-Cre+ | 20-29 | 26-29 | 20-29 |
| Number of missing values | | 36 | 38 | 80 |

**Supplemental Table 3. Data summary for behavioral tests used for mixed-effects model analysis.**
